# Precision medicine and Treat-to-Target approach in atopic dermatitis: enhancing personalized care and outcomes^☆^

**DOI:** 10.1016/j.abd.2025.501135

**Published:** 2025-06-18

**Authors:** Dan Hartmann, Catalina Retamal, Fernando Valenzuela

**Affiliations:** a Department of Dermatology, Faculty of Medicine, Universidad de Chile, Santiago, Chile; b Department of Dermatology, Clínica Universidad de los Andes, Santiago, Chile

**Keywords:** Dermatitis, atopic, Epigenomics, Multiomics, Precision medicine, Proteomics

## Abstract

**Background:**

Atopic Dermatitis (AD) is a chronic inflammatory skin disease affecting a significant portion of the global population. While conventional treatments effectively control and manage symptoms, there is a growing need for more personalized and precise approaches for patients. Precision medicine has emerged as a promising therapeutic strategy that tailors treatment to the individual characteristics of each patient. Complementing this, the Treat-to-Target (T2T) strategy sets specific clinical goals and involves continuous monitoring and treatment adjustments based on real-time patient responses and biomarker data.

**Objectives:**

This review aims to explore the latest advancements in precision medicine and the T2T strategy for AD.

**Methods:**

A comprehensive literature review was conducted to identify the most recent studies and advancements in precision medicine and the T2T strategy for AD.

**Results:**

Recent studies highlight the effectiveness of precision medicine in achieving sustained remission and improving the quality of life for patients. The T2T strategy was shown to be effective in preventing exacerbations and adapting treatments based on real-time patient responses and biomarker data.

**Study limitations:**

The lack of a consensus on the clinical implementation of precision medicine and T2T, limited longitudinal studies validating long-term outcomes, challenges in stratifying patients due to biomarker heterogeneity, and barriers to integrating emerging diagnostic technologies into routine practice.

**Conclusions:**

Precision medicine and the T2T strategy represent significant advancements in the management of AD. These approaches offer the potential for more personalized, effective, and adaptive treatment protocols, leading to improved patient outcomes. Continuous research and development in these areas are essential to fully realize their potential and integrate them into routine clinical practice.

## Introduction

Atopic dermatitis (AD) is a chronic, recurrent, and multifactorial inflammatory skin disease characterized by the presence of pruritus, eczema, erythema, and xerosis. It affects people of all ages, up to 20% of children and 10% of adults in developed countries,^1^ and it’s associated with a significant impact on patients’ quality of life. It is caused by a complex interaction of genetic, immunological, and environmental factors, leading to an alteration of the immune system that triggers inflammatory processes mainly mediated by T-cells. Among the main pathophysiological mechanisms are the disruption of the skin barrier, increased serum IgE levels, and altered synthesis of skin barrier components such as filaggrin (FLG).^2^

The family history of AD has been described as the most important disease risk factor,^1^ and it is estimated that the heritability of AD is approximately 80%,^3^ highlighting the significant importance of genetic factors in this condition. Furthermore, the identification of specific disease biomarkers is crucial to understanding the pathophysiology of AD, and these biomarkers can include genetic profiles, immunological markers, and microbiome characteristics of the skin. Multiple molecules have been identified in mechanisms contributing to skin barrier damage, FLG alteration or synthesis of loricrin, changes in membrane lipid metabolism such as ceramides, loss of anchoring junction integrity, and dysregulation of the keratinocyte differentiation process.^4^ Alterations in the microbiome of AD patients have also been identified, with a reduction in commensal bacteria and an increase in the colonization of *Staphylococcus aureus* (SA) and *Streptococcus epidermidis* (SE).^5^, ^6^ The detection and quantification of these biomarkers can provide valuable information on disease severity, treatment response, and the identification of potential therapeutic targets.

Conventional treatments for AD include topical corticosteroids and emollients, which have been the cornerstone of disease management; however, there is considerable variability in patients’ responses to these therapies. Moderate to severe AD has traditionally been treated with a variety of therapeutic options, including methotrexate (MTX), phototherapy, and cyclosporine A (CsA). In recent years, the therapeutic landscape has evolved with the incorporation of new biological and targeted treatments, such as dupilumabe and Janus Kinase (JAK) inhibitors.^7^ These new therapies have shown promising results in improving symptoms and patients' quality of life.^8^ However, significant challenges remain in clinical practice, particularly in determining which treatment is most appropriate for each patient and at what specific stage of their disease.^9^ Current treatment guidelines provide general recommendations but still lack sufficient clarity and consensus regarding the personalization of treatment based on individual patient characteristics. This uncertainty highlights the need for a more precise and personalized approach to managing moderate to severe AD.

In this context, precision medicine emerges as a new perspective in the treatment of AD, allowing a more individualized approach tailored to the genetic, biomolecular, and environmental characteristics of each patient (Table 1). This review aims to explore the latest advancements in precision medicine and the T2T strategy for AD.

**Table 1:** State-of-the-art of the knowledge about Precision Medicine and Treat-to-Target therapy in atopic dermatitis.

Precision Medicine	Treat-to-Target (T2T)
Is the treatment based on individual genetic, molecular and environmental characteristics.	T2T is a set of specific clinical goals for remission or disease reduction with regular monitoring and adjustments.
The goal is optimizing therapy effectiveness and personalize treatment to specific patient profile.	The goal is to achieve clinical goals through defined steps, constant evaluations, and treatment adjustments.
The techniques correspond to the omics sciences, focused on the genetic insights in atopic dermatitis (FLG, SPINK5, RETN, IL-13, TSLP, among others). Tools: GWAS, NGS and PRS.	Use of validated measures (e.g., EASI, Pruritus NRS, DLQI) to assess treatment effectiveness and adjust therapy.
Biomarkers focused on treatment response (IL-22, IL-16) and prognostic (CCL22 levels in the skin).	Framework for systematic decisions (e.g., increasing dose, adding therapies, or switching treatment).
It can be applied to predictive tools (e.g., Mind.Px™ patch) to determine patient response to biologics and stratification based on cytokine profiles (e.g., Th1/Th2/Th17 dominance).	It focusses on the integration of outcome measures and patient collaboration for better adherence and shared decision-making.
Diagnostic tools like RNA microneedle patches and gene expression profiling for non-invasive diagnosis and therapy optimization.	Adjust therapies based on treatment response criteria, transitioning from flare control to remission.
The challenges are the translating omics-based findings into clinical practice, and the higher costs and accessibility of technologies.	The challenges are the lack of universal consensus for dermatological T2T approaches, and the need for multidisciplinary agreements.
We still need the validation of biomarkers for therapy and prognosis, and more studies on response profiles for different endotypes.	The T2T still need the development of standardized criteria and tools to the correct implement of T2T broadly in atopic dermatitis.

## Material and methods

During August and October 2024, the authors conducted a narrative review of the literature by entering the terms "precision medicine," "atopic dermatitis," "treat-to-target therapy," "dermatology," "omics," "epigenomic," and "proteomic" into PubMed and Google Scholar. The search was limited to articles in English and Spanish. All three authors participated in the search and subsequently selected the articles based on their relevance.

## Results

### Precision medicine

The term “precision medicine” refers to the concept of tailoring the treatment and prevention of diseases by considering the different genetic, environmental, or lifestyle factors specific to groups of people.^10^ Currently, precision medicine uses the genetic and molecular information of a group of patients to develop specific and optimized medications or treatments; its primary goal is to ensure that each medication or treatment is the most suitable for treating an individual, focusing also on a reduction of side effects and greater efficacy.^11^

Advances in new technologies in the areas of molecular and structural biology, also known as omics, will play a key role in understanding the pathophysiological mechanisms of this disease and could develop preventive strategies and personalized treatments.^1^, ^12^ Precision medicine primarily focuses on human genetic information. Genetics involves the study of genes, their effects, and their relationship with inheritance. Within these areas are the "omics" sciences, which include: genomics, transcriptomics, proteomics, metabolomics, glycomics, lipidomics, epigenomics, metallomics, metagenomics, among others^11^, ^13^ (Table 2). For the study of these omics and measurement of biomarkers, there are different techniques of translational immunologic approaches to measuring biomarkers in inflammatory skin conditions such as tape stripping, microneedle-based dermal biomarker patch, molecular profiling form epidermal curettage, RNA in situ hybridization staining, single-cell RNA sequencing,^14^ suction blistering^15^ and skin microdyalisis^16^ (Table 3).

**Table 2:** Omics sciences use in medicine.

Omics	Study	T	Detection
Genomics	The study of all the genes of a person (genome), including gene interactions with each other and their environment.	DNA	GWAS, NGS
Transcriptomics	The study of the transcriptome, the quantity, and activities of all RNAs (coding and non-coding), at a given time and place.	mRNA	NGS
			Microarrays, RNA-seq
Proteomics	The study of the proteins of an organism, tissue type, or cell (proteome).	Proteins	Matrix-Assisted Laser Desorption/Ionization Time-of-Flight Mass Spectrometry (MALDI-TOF-MS) and Liquid Chromatography Coupled with Tandem Mass Spectrometry (LC-MS/MS).
Metabolomics	The study of small molecules (metabolites) within tissues, biofluids, or cells; and their interactions within a biological system (metabolome). It reflects the underlying biochemical activity and state of cells or tissues.	Metabolites	Mass Spectrometry (MS)
Glycomics	The study of carbohydrates within a cell or tissue.	Carbohydrates	Mass Spectrometry (MS)
Lipidomics	The study of lipids within a cell or tissue.	Lipids	Mass Spectrometry (MS)
Epigenomics	The study of the complete set of epigenetic modifications on the genetic material of a cell (epigenome).	DNA	ChIP-Seq/Hi-C/ChIA-PET
Metallomics	The study of metalloproteins, metalloids, and metals within a cell or tissue. It is a branch of metabolomics.	Metalloproteins, metalloids, and metals.	Atomic Absorption Spectroscopy (AAS), Inductively Coupled Plasma Mass Spectrometry (ICP-MS)
Metagenomics	The study of the genomes of a microbial community.	DNA/rRNA	NGS

**Table 3:** Investigational approaches for precision medicine in atopic dermatitis.

	Tape Stripping	Microneedle-based dermal Biomarker Patch	Molecular profiling from epidermal curettage	RNA in situ hybridation staining	Single cell RNA sequencing	Suction Blistering	Skin microdyalisis
Tecnique	Adhesive tape strips are repeatedly applied to a patient’s skin	Micron-sized needles sample RNA or protein from the skin	Curretting superficial epidermal tissue	Detects specific mRNA molecules by hybridation of complementary 18‒25bp probes and using a chromogeni or fluorescent molecule	Transcriptomic analysis of individual cells in tissues	Blisters are induced with negative pressure in the dermo-epidermal junction. The blister fluid is used for proteomic analysis and cells for transcriptomic analysis.	A microdialysis probe tip is inserted at the dermoepidermal junction and the membrane is perfused with physiological solution and molecules in the dialysate are collected.
Layers of skin obtained	Superficial epidermis	Epidermis and upper Dermis	Varies depending on anatomical location	Epidermis and dermis	Epidermis and dermis	Epidermis and upper dermis	Not reported
Sequencing analysis	Yes	Yes	No	No	Yes	Yes	No
Biopsy needed	No	No	No	Yes	Yes	No	No
Limitations	Predominantly samples stratum corneum and difficulties standardizing	Less invasive than biopsy and the techinique could lead to irritation and discomfort	There is no data reported on the reproducibility of the collection method	Difficulty of analyzing large cytokine panels since each target requires a separate probe	Difficulty in integration into the clinical workflow and samples may need to be processed fresh.	Time required for blistering formation (1 hour)	The collection of molecules varied with body temperature, blood flow and perfusion rate. It is difficult to standardize these factors.

### Genetics in atopic dermatitis

Precision medicine recognizes the importance of individual factors such as genetics, medical history, and the patient’s environment in the development and management of AD. Personalized approaches can include selecting specific treatments based on genetic profiles, identifying and avoiding environmental triggers, and regularly monitoring treatment responses using specific biomarkers. The implementation of personalized strategies can significantly improve treatment efficacy and tolerability, as well as reduce the risk of unwanted side effects.

As previously mentioned, genetics plays a key and fundamental role in the etiopathogenesis of AD. The first genetic linkage study, which compared the inheritance of genetic markers with the presence of a clinical trait to identify AD risk genes, identified an important susceptibility locus on chromosome 3q21.^17^ Additionally, the *FLG* gene, which encodes filaggrin, is one of the best-known genes associated with AD susceptibility and severity.^18^ A similar association has been found with another mutation in the serine protease SPINK5.^19^, ^20^ Studies conducted in Asian populations identified an association between the SPINK5 gene, which encodes a serine protease inhibitor involved in maintaining the skin barrier, and AD susceptibility.^21^

In recent years, multiple genetic risk factors related to AD have been identified, including ADAM33, RETN, TGFB1, CD207, TSLP, IL-13, IL-4, IL-31, IL6R, STAT3, KIF3A, and HLA-DBQ1, among others^13^, ^22^, ^23^, ^24^, ^25^, ^26^, ^27^ (Table 4). Genetic risk factors for AD have been identified through genetic linkage studies, candidate gene approaches, Genome-Wide Association Studies (GWAS), and more recently, Next-Generation Sequencing (NGS) technologies, which have contributed to a better understanding of the genetic risk basis of AD.^1^

**Table 4:** Genes associated with atopic dermatitis.

Gene	Location	Function	Reference
Filagrin	Chromosome 1q21.3	Part of the epidermal differentiation complex. Skin barrier development and maintenance.	(Løset M, 2019) ^29^
SPINK5	Chromosome 5q32	Protease inhibitor for epidermis homeostasis	(Bin L; 2016) ^28^
ADAM33	Chromosome 20p13	Cell-cell and cell-matrix interactions, cell migration and cell adhesion	(Matsuse A; 2009) ^24^
TSLP	Chromosome 5q22.1	Promotes Th2 immune response	(Bin L; 2016) ^23^
IL-4	Chromosome 5q31	Part of the Th2 signaling pathway	(Bin L; 2016) ^23^
IL-13	Chromosome 5q31	Part of the Th2 signaling pathway	(Bin L; 2016) ^23^
IL-31	Chromosome 5q31	Part of the Th2 signaling pathway	(Bin L; 2016) ^23^
IL6R	Chromosome 1q21.3	Immune response in multiple axes	(Nakajima S; 2024) ^13^
STAT3	Chromosome 17q21.2	JAK-STAT signalling in multiple immune axes	(Nakajima S; 2024) ^13^
HLA-DBQ1	Chromosome 6p21.32	Antigen presentation	(Nakajima S; 2024) ^13^
CD207	Chromosome 2p13.3	Activation of Langerhans cells	(Nakajima S; 2024) ^13^
KIF3A	Chromosome 5q31	Formation of motile and nonmotile primaria cilia. Associated to asma and atopic march.	(Stevens ML; 2020) ^25^
RETN	Chromosome 19p13	Promotes expresión of pro-inflammatory cytokines	(Banihani SA; 2018) ^27^
TGFB1	Chromosome 19q13.2	Modulates inflammation and tissue remodeling	(Shafi T; 2024) ^26^

GWAS is a statistical approach that compares the allelic frequency of millions of genetic variants between patients and healthy subjects to identify loci associated with clinical traits.^28^ These studies have identified more than 40 genes associated with AD risk, including the FLG gene, and risk loci such as SERPINB7, DSC1, and ITGB8, among others.^1^ In phenotype-wide association studies, a loss-of-function variant of FLG is associated with different clinical phenotypes of AD, such as asthma, allergic rhinitis, and food allergy.^29^ Genetic variation in the *MMP ADAM33* genes and the CD14 monocyte receptor has also been associated with allergic bronchitis and asthma, respectively.^30^

Next-generation Exome Sequencing (WES) allows the identification of genetic variation in exomic regions, enabling the characterization of genetic factors underlying AD.^1^ Rare variants in *FLG* and other genes such as *GTF2H5*, *EVPL*, or *NLRP1* have been identified in African populations.^31^ Another study identified a mutation in *CARD11* that generates potentially correctable cellular alterations leading to AD.^32^

Among the new advances in genetic studies, Polygenic Risk Scores (PRS) have emerged. These scores are being used to identify patients at risk of developing AD and who are more likely to benefit from early therapeutic intervention. PRS is a quantitative measure that estimates an individual’s genetic risk burden for developing a specific disease, calculated by aggregating the risk conferred by multiple small-effect variants distributed throughout the genome.^1^

### Applications of precision medicine

Recently, the development of non-invasive diagnostic tools for AD and psoriasis has helped with different conditions and guided effective therapies. Additionally, patients often experience frustration due to trial-and-error treatment approaches, which delay disease control and impact quality of life. Two companies, Mindera Health and Castle Biosciences, are pioneering non-invasive technologies to address these challenges.^33^ Mindera Health developed the Mind.Px™ dermal patch, which uses microneedles to collect RNA from skin samples. Machine learning and genetic biomarkers predict patient responses to specific psoriasis biologics, reducing trial-and-error treatments.^34^ Clinical studies (e.g., STAMP-1, STAMP-2, MATCH) demonstrated its economic and clinical benefits, showing high predictive accuracy in severe psoriasis cases, and reducing biological therapy costs.^33^ On the other hand, Castle Biosciences uses non-invasive skin scrapings and gene expression profiling to distinguish between psoriasis, AD, and other conditions like mycosis fungoides.^33^ Preliminary studies identified distinct genetic markers linked to treatment responses in patients using drugs like dupilumab and risankizumab.^35^ Ongoing clinical trials aim to validate these findings and optimize systemic therapy selection. These companies aim to revolutionize precision medicine in dermatology by improving diagnosis and tailoring treatment based on genetic profiles, but there still challenges to resolve, including insurance coverage, integration into clinical workflows, and patient acceptance. Successful implementation of these technologies could significantly reduce the burden of inflammatory skin diseases.

Proteomics has been useful in finding biomarkers for both treatment response and prognosis. Studies have observed elevated levels of various inflammatory protein markers in both lesional skin and serum of patients, including proteins related to immune response activation and associated with Th1, Th2, Th17, and Th22 responses.^36^ These methods have also been used to identify treatment response markers, measuring levels of chemokines and cytokines such as IL-22 and IL-16 before and after treatment with topical corticosteroids, showing a decrease in observed levels, and even correlating the decrease in IL-16 with clinical improvement after topical treatment.^37^, ^38^ In 2023, Bakker et al. conducted a review of various studies that attempted to classify AD patients based on their immunologic biomarkers.^39^ Among the existing studies to date, Thijs et al. and Bakker et al., in different cohorts identified the following endotypes (Table 5): Dominant Th1/Th2/Th17 Profile, Dominant Th2/Th22/PARC Profile, and Dominant Th2/eosinophils Profile. The first two groups showed high levels of IL-4, IL-5, and IL-13 expression.^40^, ^41^

**Table 5:** Bakker endotypes of atopic dermatitis.^41^

Endotype	Immune Pathway	Biomarkers	Clinical Characteristics	Treatment Implications
Th1/Th2/Th17 Dominant Profile	Th1, Th2, Th17	IL-4, IL-5, IL-13, IL-17.	Mixed immune activation; observed in some cohorts; associated with inflammation and barrier dysfunction.	Dupilumab (anti-IL-4/IL-13), Tralokinumab (anti-IL-13), and Lebrikizumab (anti-IL-13).
Th2/Th22/PARC Dominant Profile	Th2, Th22	IL-4, IL-5, IL-13, PARC (CCL18).	High allergic response, skin thickening (lichenification); linked to chronic AD lesions.	Dupilumab (anti-IL-4/IL-13), Tralokinumab (anti-IL-13), Lebrikizumab (anti-IL-13).
Th2/Eosinophils Dominant Profile	Th2	IL-4, IL-5, IL-13, Eosinophils.	Strong allergic sensitization with significant eosinophilia; typical of severe allergic phenotypes.	May require broader treatments like JAK inhibitors (Upadacitinib, Abrocitinib, Baricitinib) due to complex immune involvement.

For this reason, it has been suggested that they could be good candidates for targeted therapies such as dupilumab (anti-IL-4/IL-13 monoclonal antibody), tralokinumab (anti-IL-13), and Lebrikizumab (anti-IL-13),^42^, ^43^ while patients in the remaining group may require drugs with a broader action profile, such as JAK inhibitors (upadacitinib, abrocitinib, baricitinib).^39^, ^41^ However, more studies are still needed to evaluate the different response profiles of each of these endotypes to the existing therapeutic options.

Thus, the authors can define 2 endotypes based on cytokine expression, referred to by Dyjack et al. as Type 2-predominant inflammatory response endotype and non-Type 2-predominant inflammatory response endotype^44^ (Table 6). In turn, cytokine-based endotypes can be stratified by patient age. It has been observed that children with early-onset AD have a Th2/Th22/Th17 predominance,^45^, ^46^ with a lack of Th1, while adults have a Th22 predominance.^47^ It has also been observed that adolescents have higher levels of IL-22.^48^

**Table 6:** Th2 predominant and Non-Th2 predominate endotypes of atopic dermatitis.^44^

Type Th2-Predominant Endotype	Non-Type Th2-Predominant Endotype
This endotype is driven primarily by Th2-mediated cytokines: IL-4, IL-13, and IL-31.	This subtype involves Th1, Th17, and Th22 pathways alongside Type 2 responses, especially in specific patient populations or chronic phases.
It is associated with acute inflammation, itch, and barrier dysfunction due to the suppression of antimicrobial peptides and proteins like filaggrin.	Cytokines such as IL-17, IL-22, and IFN-γ are more pronounced in this group.
Treatments like Dupilumab (anti-IL-4/IL-13) and other biologics targeting Type 2 cytokines are effective in managing this subtype.	It is often observed in older patients or those with more complex AD presentations.
This form is prevalent in most patients with AD, including children and those with early-onset disease.	Broader immunomodulators, including JAK inhibitors, may be needed for treatment due to the overlap of pathways.

Regarding prognostic biomarkers, CCL22 expression levels in the skin have been identified as a marker that predicts clinical improvement during the use of various targeted therapies such as topical crisaborole, CsA, and fezakinumab (anti-IL-22 monoclonal antibody).^49^

### Targeted treatment or Treat-to-Target (T2T)

In managing patients with AD, decision-making for selecting treatment and guiding evaluation in individual patients can be complex and often subjective.^50^ The introduction of highly effective drugs has led to the development of treatment algorithms based on specific treatment response targets, using algorithms to guide decisions to continue, discontinue, or modify treatment.^51^ The “Treat-to-Target” (T2T) strategy is defined as a targeted treatment (towards remission or reduction of disease activity) and the application of strict controls (scheduled visits with respective treatment adjustments) to achieve a proposed goal.^52^ Another definition of T2T could be the therapeutic strategy of defining treatment with a specific objective through defined steps with constant evaluations to determine if the proposed objectives have been achieved.^53^

This approach provides a framework to support treatment options made using shared clinical information, and decision-making with patients, including discussion of relevant risk/benefit issues of existing or alternative treatments.^50^ While T2T is well-defined in other inflammatory/immunological pathologies, there are still no clearly established consensuses for dermatological diseases. However, T2T is highly relevant in AD, which represents a therapeutic challenge due to its heterogeneous clinical presentation and significant impact of symptoms on quality of life, especially pruritus.^54^

A recent international consensus on T2T in AD determined that patients should be evaluated with validated measures such as the Eczema Area and Severity Index (EASI), the Maximum Pruritus Numeric Rating Scale, the Dermatology Life Quality Index (DLQI), and the Patient-Oriented Eczema Measure at 3-and 6-months, using a T2T strategy. The Committee combined clinical experience with available recommendations and guidelines, including those from Harmonizing Outcome Measures for Eczema (HOME), to develop practical T2T criteria. A unanimous agreement was reached to evaluate patients with at least 1 of 2 physician-assessed outcome measures (EASI and Physician's Global Assessment) and at least 1 of 3 patient-reported outcome measures (Pruritus Numeric Rating Scale, DLQI, and Patient-Oriented Eczema Measure). With 3 medical visits per year to achieve the ideal therapeutic transition from flare control to induction and maintenance of remission. A treatment will be considered effective if at least one physician-assessed outcome goal and at least one patient-reported outcome goal are met. If target criteria are not met, modifications should be made to optimize the current treatment (e.g., increasing the dose or frequency of systemic therapy), adding adjunctive treatment (e.g., phototherapy or another systemic therapy), or changing the current treatment.^51^

In conclusion, although the T2T approach can become a useful tool to simplify therapeutic goals and management of AD, its foundation is just beginning to be built. A multidisciplinary approach, including a wide range of stakeholders, including patients, is needed to better define the essential components necessary to use T2T in AD.^55^

## Discussion

Precision medicine represents a new era in the treatment of AD, marking a transition from the “one drug fits all” approach, where treatments are stratified and defined based on the severity of the disease, to a more personalized view. This approach considers that it is unlikely for all patients to have the same response to treatments, so therapeutic decisions are based on the specific endotype of each patient, adapting to the biological characteristics and needs of the patient.

Moreover, precision medicine appears to be the way forward for guiding this diagnostic and therapeutic process with the T2T strategy. Unlike clinical phenotypes, which may not be useful for predicting the course of the disease,^56^ considering the biological, genetic, and molecular individuality of each patient allows us to predict the response to each treatment and thereby achieve the desired goal. This is especially relevant in current times, with the introduction of new systemic treatments, including biological drugs and JAK inhibitors for the treatment of atopic dermatitis. Stratifying patients based on their predominant immunological biomarkers will enable us to use existing therapies more efficiently and targeted in patients with a specific endotype where they will achieve better results.^39^, ^57^ This not only maximizes therapeutic outcomes in patients requiring these therapies, who often have moderate to severe cases that have not responded to other treatments previously,^50^ but also allows for better use of these resources.

It is important to consider that precision medicine emerges as a response to the need for a more personalized approach to the treatment of atopic dermatitis. However, to integrate its use into daily clinical practice, it is still necessary to develop various studies. These studies should aim to validate the existing findings and evaluate the clinical response and the levels of response markers (IL-22 and IL-16), and prognostic markers (CCL22) proposed within different patient groups using the existing therapies. This way, therapeutic algorithms can be developed based on this approach, and scientific findings will translate into tangible benefits for patients.^39^, ^57^

## Conclusion

Precision medicine represents a new era in the treatment of AD, offering more personalized approaches tailored to the individual needs of each patient. The identification of specific biomarkers, the development of targeted therapies, and the application of personalized approaches are key aspects in the successful implementation of precision medicine in AD. T2T corresponds to a tool that should be agreed upon by local expert groups, with objectives that are modified over time, and that allow making decisions with the patient. However, further research is needed to validate these approaches and translate scientific findings into tangible clinical benefits for patients.

## Authors’ contributions

Dan Hartmann: Approval of the final version of the manuscript; critical literature review; manuscript critical review; preparation and writing of the manuscript

Catalina Retama: Altbir: Critical literature review; manuscript critical review; preparation and writing of the manuscript.

Fernando Valenzuela: Approval of the final version of the manuscript; critical literature review; effective participation in research orientation; manuscript critical review; preparation and writing of the manuscript.

## Financial support

This research has not received specific support from public sector agencies, the commercial sector, or non-profit entities.

## Conflicts of interest

None declared.
